# Supplementary material for: Exploring the Effect of Functional Diets Containing Phytobiotic Compounds in Whiteleg Shrimp Health: Resistance to Acute Hepatopancreatic Necrotic Disease Caused by Vibrio parahaemolyticus
Source: Animals (Basel). 2023 Apr 15;13(8):1354. doi: 10.3390/ani13081354 (PMC10135097; doi:10.3390/ani13081354)
Supplement: Supplementary file 1 [file animals-13-01354-s001.zip › animals-2319543-supplementary.pdf]

Table S1. Bacterial strains used in this study.

Strain	Species	Origin	*pir*A/ *pir*B genes	Reference
*Vp*AHPND	*Vibrio parahaemolyticus*	Diseased shrimp (Taiwan)	+	[18]
Vp lab1	*V. parahaemolyticus*	Diseased shrimp (Ecuador)	+	Lab collection (not published)
Vp lab2	*V. parahaemolyticus*	Diseased shrimp (Vietnam)	+	Lab collection (not published)
CECT 8407	*V. parahaemolyticus*	Diseased shrimp (Mexico)	-	[64]
Valg lab1	*V. alginolyticus*	Healthy European seabass	-	Lab collection (not published)
CECT 8408	*V. harveyi*	Lesion of shrimp (Mexico)	-	[65]
CECT 4999	*V. vulnificus*	Diseased eel (Spain)	-	[66]

Table S2. Primers tested for *Vibrio parahaemolyticus* qPCR optimization.

Target gene	Primer	Primer sequence (5′- 3′)	Reference
tlh	Tlh_F3	GCTACTCGAAAGATGATCC	[67]
	Tlh_LB	TAGATTCGAACGAGA	
tdh	D3	CCACTACCACTCTCATATGC	[68]
	D5	GGTACTAAATGGCTGACATC	
tlh	Tlh-Fw	AACTTCTGCGCCCGAAGAG	[69]
	Tlh-Rv	CGGTGGATGTCCAAACAAGGA	
tdh	Tdh-Fe	GTAAAGGTCTCTGACTTTTGGAC	[67]
	Tdh-Rv	CTACAGAATCATAGGAATGTTGAAG	
trh	Trh-Fw	CCATCAATACCTTTTCCTTCTCC	[67]
	Trh-Rv	ACCGTCATATAGGCGCTTAAC	
pirA	PirA-Fw	TTGGACTGTCGAACCAAACG	[50]
	PirA-Rv	GCACCCCATTGGTATTGAATG	
tlh	VPF	AACCGTGGCGTTCCAGAA	[49]
	VPR	CCGTCAAACGAATCAGTGCTT	

Table S3. Detection of *Vibrio parahaemolyticus* strains by qPCR assay comparing PirA-Fw/PirA-Rv and VPF/VPR primer pairs.

V. parahaemolyticus strain	CT (qPCR PirA-Fw/PirA-Rv)	CT (qPCR VPF/VPR)
*Vp*AHPND	14.89	12.32
Vp lab1	17.82	14.81
Vp lab2	16.31	14.42
CECT 8407	34.56	15.44

References

[18] Lee, C.; Chen, I.T.; Yang, Y.T.; Ko, T.P.; Huang, Y.T.; Huang, J.Y.; Huang, M.F.; Lin, S.J.; Chen, C.Y.; Lin, S.S.; Lightner, D.V.; Wang, H.C.; Wang, A.H.J.; Wang, H.C.; Hor, L.I.; Lo, C.F. The opportunistic marine pathogen

Vibrio parahaemolyticus becomes virulent by acquiring a plasmid that expresses a deadly toxin. *PNAS,* **2015**, 112 (34), 10798–10803. doi: 10.1073/pnas.1503129112.

[49] Park, J.Y.; Jeon, S.; Kim, J.Y.; Park, M.; Kim, S. Multiplex Real-time Polymerase Chain Reaction Assays for Simultaneous Detection of *Vibrio cholerae*, *Vibrio parahaemolyticus*, and *Vibrio vulnificus*. *Os Pub Health Res Pers,* **2013**, 4 (3),133–139. doi: 10.1016/j.phrp.2013.04.004.

[50] Han, J.E.; Tang, K.F.J.; Tran, L.H.; Lightner D.V. Photorhabdus insect-related (Pir) toxin-like genes in a plasmid of *Vibrio parahaemolyticus*, the causative agent of acute hepatopancreatic necrosis disease (AHPND) of shrimp. *Dis AquatiC Org*, **2015**, l (113), 33–40. doi: 10.3354/dao02830.

[64] Roque, A.; Turnbull, J.F.; Escalante, G.; Gomez-Gil, B.; Alday-Sanz, M.V. Development of a bath challenge for the marine shrimp *Penaeus vannamei* Boone, 1931. *Aquaculture,* **1998**, 169, 283–290. doi: 10.1016/S0044-8486(98)00370-6.

[65] Soto-Rodriguez, S.A.; Gomez-Gil, B.; Lozano, R.; del Rio-Rodríguez, R.; Diéguez, A.L.; Romalde, J.L. Virulence of *Vibrio harveyi* responsible for the "Bright-red" Syndrome in the Pacific white shrimp *Litopenaeus vannamei*. *J Invertebr Pathol*. **2012**, 109 (3), 307-317. doi: 10.1016/j.jip.2012.01.006.

[66] Lee, C.T.; Amaro, C.; Wu, K.M.; Valiente, E.; Chang, Y.F.; Tsai, S.F.; Chang, C.H.; Hor, L.I. A common virulence plasmid in biotype 2 *Vibrio vulnificus* and its dissemination aided by a conjugal plasmid. *J. Bacteriol.* **2008**, 190, 1638–1648. doi: 10.1128/JB.01484-07.

[67] Wang, H.; Tang, X.; Su, Y.C.; Chen, J.; Yan, J. Characterization of clinical *Vibrio parahaemolyticus* strains in Zhoushan, China, from 2013 to 2014. *PLoS ONE*. **2017**, 12 (7), e0180335. doi: 10.1371/journal.pone.0180335

[68] Yamazaki, W.; Kumeda, Y.; Misawa, N.; Nakaguchi, Y.; Nishibuchi, M. Development of a loop-mediated isothermal amplification assay for sensitive and rapid detection of the *tdh* and *trh* genes of *Vibrio parahaemolyticus* and related *Vibrio* species. *Appl Environ Microbiol*. **2010**, 76 (3), 820-828. doi: 10.1128/AEM.02284-09.

[69] Kaufman, G.E.; Blackstone, G.M.; Vickery, M.C.; Bej, A.K.; Bowers, J. Bowen, M.D.; Meyer, R.F. DePaola, A. Real-time PCR quantification of *Vibrio parahaemolyticus* in oysters using an alternative matrix. *J Food Prot*. **2004**, 67 (11), 2424-2429. doi: 10.4315/0362-028x-67.11.2424.
